# Targeting ADAM17 inhibits human colorectal adenocarcinoma progression and tumor-initiating cell frequency

**DOI:** 10.18632/oncotarget.17780

**Published:** 2017-05-10

**Authors:** Joseph Dosch, Elizabeth Ziemke, Shanshan Wan, Kathryn Luker, Theodore Welling, Karin Hardiman, Eric Fearon, Suneetha Thomas, Matthew Flynn, Jonathan Rios-Doria, Robert Hollingsworth, Ronald Herbst, Elaine Hurt, Judith Sebolt-Leopold

**Affiliations:** ^1^ Department of Radiology, University of Michigan, Ann Arbor, MI 48109, USA; ^2^ Department of Surgery, University of Michigan, Ann Arbor, MI 48109, USA; ^3^ Department of Internal Medicine, University of Michigan, Ann Arbor, MI 48109, USA; ^4^ Department of Oncology Research, MedImmune, LLC, Gaithersburg, MD 20878, USA

**Keywords:** ADAM17, TACE, colorectal, stem cell, Notch

## Abstract

ADAM17 (a disintegrin and metalloproteinase 17)/TACE (TNFα converting enzyme) has emerged as a potential therapeutic target in colorectal cancer (CRC) and other cancers, due in part to its role in regulating various tumor cell surface proteins and growth factors and cytokines in the tumor microenvironment. The emergence of MEDI3622, a highly potent and specific antibody-based ADAM17 inhibitor, has allowed testing of the concept that targeting ADAM17 may be an important new therapeutic approach for CRC patients. We demonstrate that MEDI3622 is highly efficacious on tumor growth in multiple human CRC PDX models, resulting in improved survival of animals bearing tumor xenografts. MEDI3622 was further found to impact Notch pathway activity and tumor-initiating cells. The promising preclinical activity seen here supports further clinical investigation of this treatment approach to improve therapeutic outcome for patients diagnosed with metastatic CRC, including patients with KRAS-mutant tumors for whom other therapeutic options are currently limited.

## INTRODUCTION

Colorectal cancer (CRC) is the third leading cause of cancer-related deaths in the United States and a major and growing health problem around the world [1]. Surgical resection remains the chief curative option for patients. However, despite advances in early detection, > 50% of CRC patients present with regional invasion and/or distant metastatic disease [2]. Conventional chemotherapeutic regimes for CRC patients with advanced disease are limited and are based on 5-fluorouracil/leucovorin treatment combined with oxaliplatin (FOLFOX) or irinotecan (FOLFIRI) combination therapy. The combinations have improved the percentage of advanced CRC patients responding to chemotherapy to 40-50% [3]. These advances in chemotherapy along with the development of targeted inhibitors, like cetuximab that inhibits the epidermal growth factor receptor (EGFR), have improved patient survival for a select group of patients. However, the overall 5-year survival rate is < 15% for patients with metastatic CRC (mCRC), highlighting the need for improved therapies.

A disintegrin and metalloprotease domain-containing protein 17 (ADAM17), also known as TNF-alpha converting enzyme (TACE) has emerged as a potential therapeutic target in CRC and other cancers. ADAM17 has potential roles in regulating various cell surface proteins on the cancer cell surface as well as growth factors and cytokines in the tumor microenvironment. Most notably, the EGFR ligands, transforming growth factor-α (TGF-α) and amphiregulin are frequently observed to be upregulated in colorectal cancer (CRC) [4–7]. ADAM17, which is required for cell surface proteolytic processing of TGF- α and amphiregulin, has been shown to be upregulated in primary and metastatic CRC tumors compared to normal colonic mucosa [8]. Inhibition of this protease with a selective small molecule inhibitor was further found to impede CRC tumor growth by targeting the EGFR axis [4]. However, non-EGFR-mediated pathways have recently been implicated for their contributions to the antitumor activity of a highly potent and specific ADAM17 inhibitory antibody [9]. The antibody, known as MEDI3622, was found to inhibit growth of COLO 205 xenografts, which were refractory to treatment with a pan-HER agent [9]. The studies described here were undertaken to evaluate the activity of MEDI3622 in a heterogeneous panel of CRC patient-derived xenograft (PDX) models, comprised of both KRAS-mutant (KRAS^MT^) and KRAS-wild type (KRAS^WT^) models. We report here that MEDI3622 exhibits significant single agent activity in multiple CRC models, including a KRAS^MT^ CRC model, and acts, in part, by targeting stem cell frequency in the tumors.

## RESULTS

### MEDI3622 suppresses growth of multiple colorectal patient-derived xenografts

**Figure 1:**
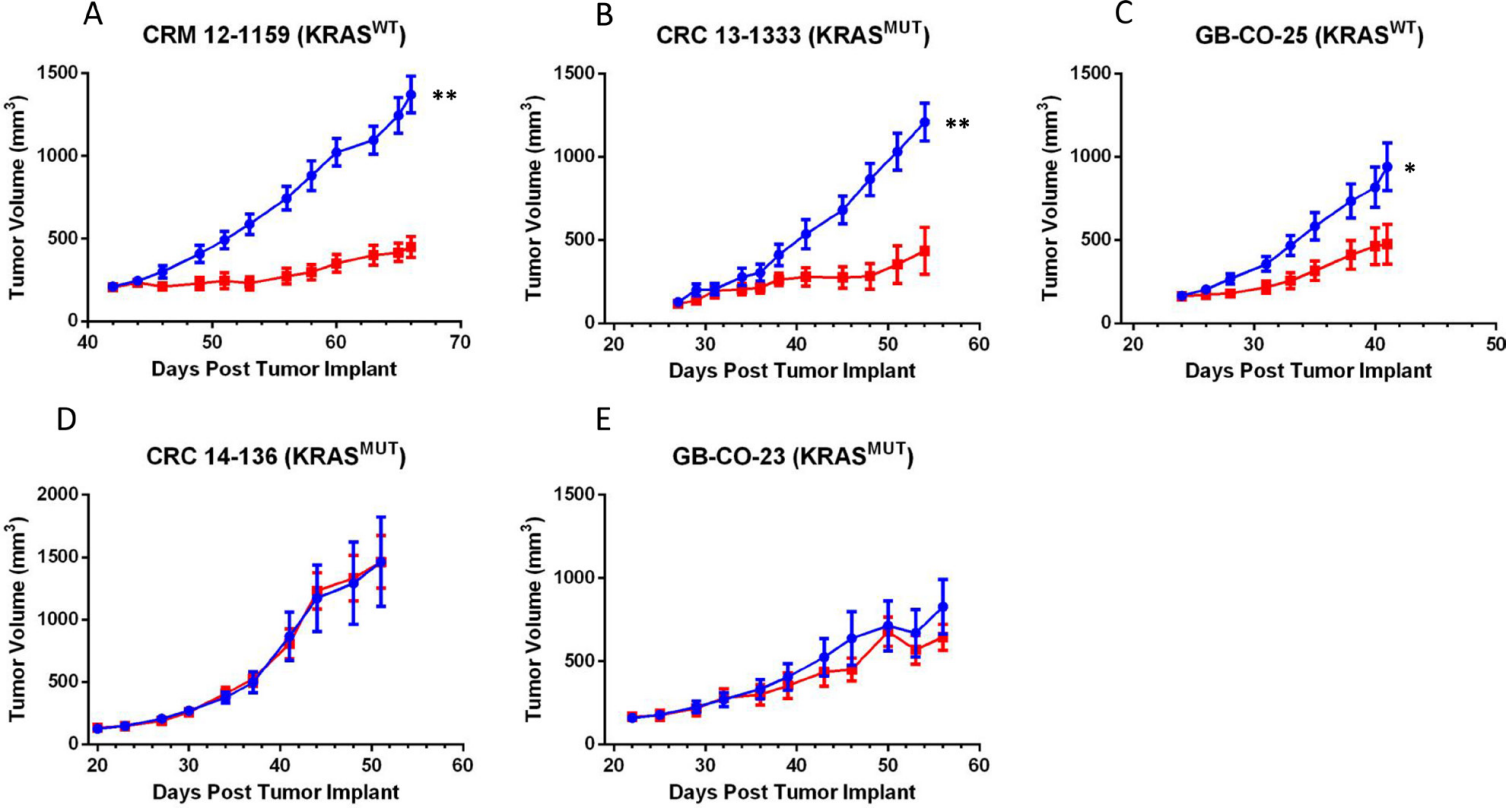
Panels (**A–E**) Comparison of the efficacy of MEDI3622 in five CRC PDX models. MEDI3622 was administered IP twice weekly for 2 to 3 weeks at a dose of 10 mg/kg in the GB-CO-23 and GB-CO-25 models and 30 mg/kg in the UM-CRM 12-1159, UM-CRC 13-1333 and UM-CRC 14-136 models. Growth of tumors in the human IgG1 control and MEDI3622-treated groups are denoted in blue and red, respectively. The number of mice in each group was eight with the exception of the CRC 13-1333 and CRC 14-136 models, where *n* = 5 and 7, respectively. Seventy-two hours after the last MEDI2622 or control treatment, tumors were excised from the mice and cryopreserved or flash frozen. An unpaired *t* test was performed to evaluate significance; ** denotes a *P* value of 0.0018 for UM-CRM-12-1159 and 0.0027 for UM-CRC-13-1333 and * denotes a *P* value of 0.0259 for GB-CO-25.

To follow up on previously published data showing that MEDI3622 was efficacious against xenografts of the established COLO 205 CRC cell line [9], *in vivo* testing of MEDI3622 was carried out in five human CRC PDX models, selected for two features: their genomic heterogeneity (Supplementary Table 1) and their maintenance of histopathological features akin to those in the primary patient cancer specimen when grown as PDX models (Supplementary Figure 1). Models designated as UM-CRM originated from metastatic lesions, whereas both GB and UM-CRC models were established from primary resected surgical specimens. TACE activity assays confirmed that all models used in the efficacy studies express the target with GB-CO-23 and GB-CO-25 having roughly half the level of activity as observed in the other three models (Supplementary Figure 2). Tumors were implanted subcutaneously and treatment was initiated when tumors reached 150–200 mg in size. Treatment consisted of either 2 or 3 cycles of IP administration (Q3Dx2) of either the human IgG1 control antibody or MEDI3622 (10 or 30 mg/kg), depending on tumor growth rate of control tumors of the particular PDX model under study. Two of the models, CRM 12-1159 and CRC 13-1333, proved to be highly sensitive to MEDI3622 as reflected by T/C values (ratio of treated/control tumor size on the last day of treatment) of 37% and 36%, respectively (Figure 1A, 1B). Another model, GB-CO-25, was moderately sensitive to treatment with this agent, as reflected by a T/C value of 51% on the last day of treatment (Figure 1C). Therefore, MEDI3622 activity was observed in PDX models that do or do not harbor a *KRAS*^*MT*^ allele. Two other KRAS^MT^ CRC PDX models, CRC 14-136 and GB-CO-23, proved to be refractory to MEDI3622 treatment (Figure 1D, 1E).

### Sensitivity to MEDI3622 correlates with Notch pathway activity and impairment of stem cell functionality

The potent activity of MEDI3622 in both the CRM 12-1159 and CRC 13-1333 PDX models indicates that MEDI3622 is active against both KRAS^WT^ and KRAS^MT^ CRCs. Two lines of evidence suggest that the anti-tumor activity of MEDI3622 is independent of EGFR activation in this panel of CRC models. First, expression of phosphorylated EGFR in models found to be refractory to MEDI3622 treatment (CRC 14-136 and GB-CO-23) showed similar pEGFR expression levels to the responsive CRC 13-1333 model (Supplementary Figure 3). Second, while CRM 12-1159 was the most sensitive model to MEDI3622 and also exhibits exceptionally high levels of pEGFR, effects of the agent on downstream signaling were not evident from evaluation of impact on pERK expression (Supplementary Figure 3). This extends the previous observation that MEDI3622 exerts it’s anti-tumor effect, in part, by modulating non-EGFR pathways [9].

**Figure 2:**
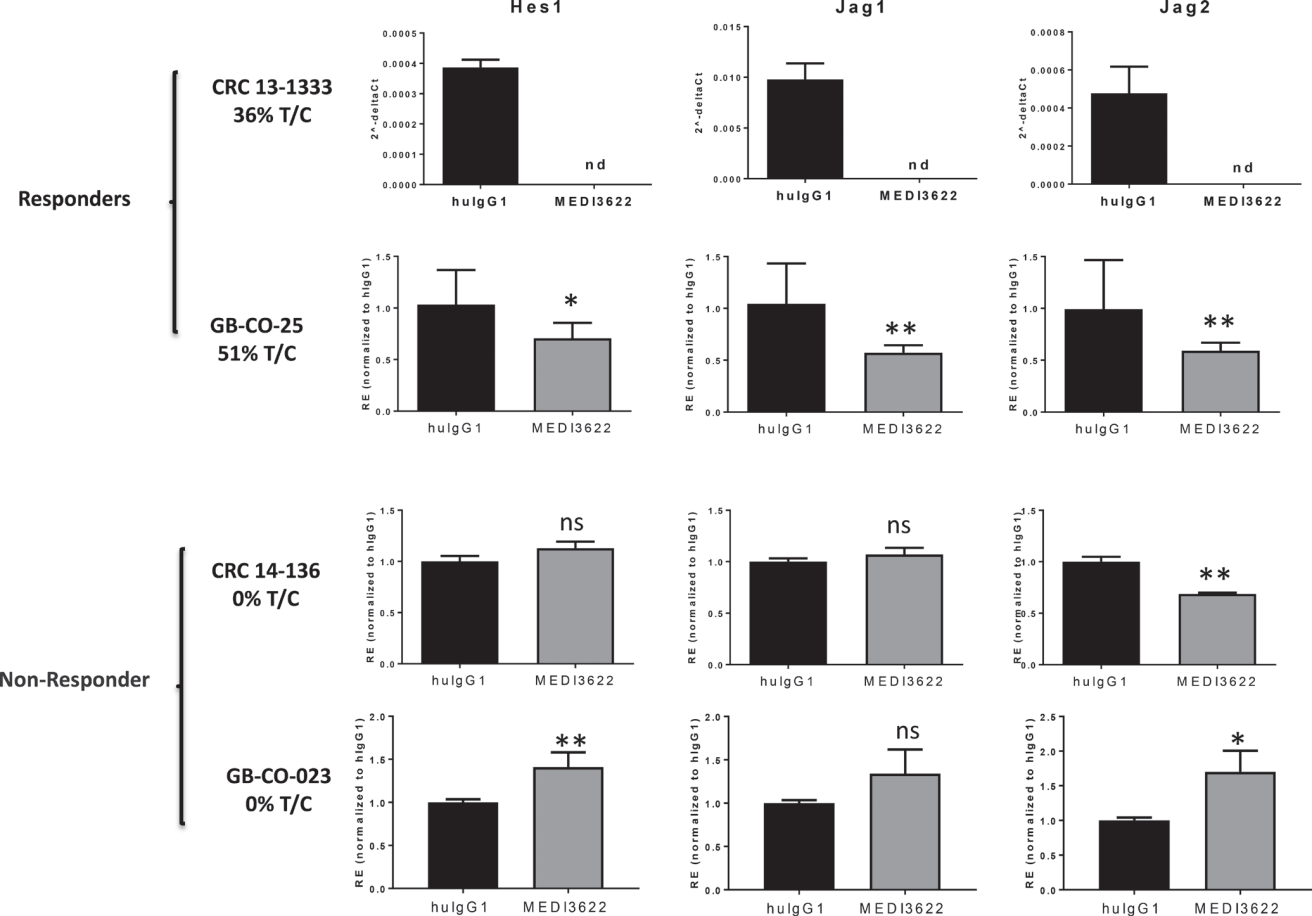
Changes in NOTCH signaling pathway genes in tumors in response to MEDI3622 treatment Total RNA was extracted from flash frozen tumor material derived from the efficacy studies in Figure 1, and quantitative gene expression analyses were carried out by RT-PCR. For CRC 13-1333 data is presented as 2^-delta CT^ due to no measurable transcript in MEDI3622 treated tumors. All other data presented as relative expression (2^-delta delta CT^). nd = not detectable and ns = not significant. A two-tailed Students’ *t* test was performed to evaluate significance and ** denotes a *P* value < 0.01 and * denotes a *P* value of < 0.05.

We therefore initiated studies to explore EGFR-independent mechanisms that might potentially contribute to the therapeutic response patterns observed in our PDX models. Based on the documented role of dysregulated Notch signaling in colon cancer [10, 11], the reported role for ADAM17 in the proteolytic cleavage of Notch family receptor and ligand molecules with effects on Notch pathway signaling [12, 13], and the ability of MEDI3622 to directly inhibit a Notch luciferase assay (Supplementary Figure 4), we evaluated gene expression levels of a subset of genes encoding Notch pathway factors. Expression of Hes1, Jag1, and Jag2 was assessed in the treated tumors from the efficacy studies by quantitative RT-PCR (Figure 2). In the CRC 13-1333 model, which was highly sensitive to MEDI3622 treatment, expression of all three genes changed from clearly detectable to non-detectable (nd) following treatment with MEDI3622. Consistent with effects on Notch pathway signaling being linked to MEDI3622 effects, the expression levels of the three Notch pathway genes were detectable but significantly reduced following MEDI3622 treatment in the moderately sensitive model GB-CO-25 CRC PDX model. In contrast, Notch pathway gene expression was completely unaffected by MEDI3622 treatment in the non-responsive models, CRC 14-136 and GB-CO-23.

**Figure 3:**
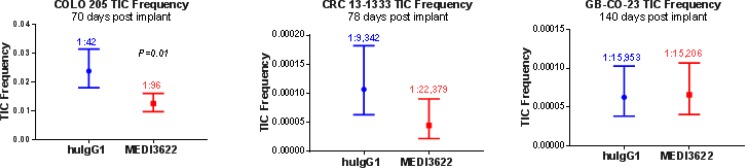
Effect of MEDI3622 treatment on tumor-initiating cell (TIC) frequency TIC frequency was measured by limited dilution analysis of cryopreserved tumor cells derived from efficacy studies. Following treatment tumors were excised, dissociated and re-implanted at various low numbers (Supplementary Table 2). The TIC frequency was calculated using L-calc software and is presented with the 95% CI frequencies shown. The *P* value for each analysis is either shown or are not significant.

Limiting dilution assays were then carried out to explore the ability of MEDI3622 to impair tumor-initiating cell (TIC) function as measured by effects on tumorigenicity. Three models were chosen for study: COLO 205, which we previously showed was responsive to MEDI3622 [9], CRC 13-1333, which also showed tumor growth inhibition with MEDI3622 treatment, and GB-CO-23, a model that was both non-responsive to MEDI3622 and did not show inhibition of the Notch signaling pathway. In the responsive models, COLO 205 and CRC 13-1333, an approximate 2-fold decrease in TIC frequency was observed. However in GB-CO-23 tumors, MEDI3622 did not inhibit the TIC population consistent with no effect on Notch signaling (Figure 3 and Supplementary Table 2).

### MEDI3622 inhibits growth of metastatic lesions arising in the liver after orthotopic surgery leading to increased survival

**Figure 4:**
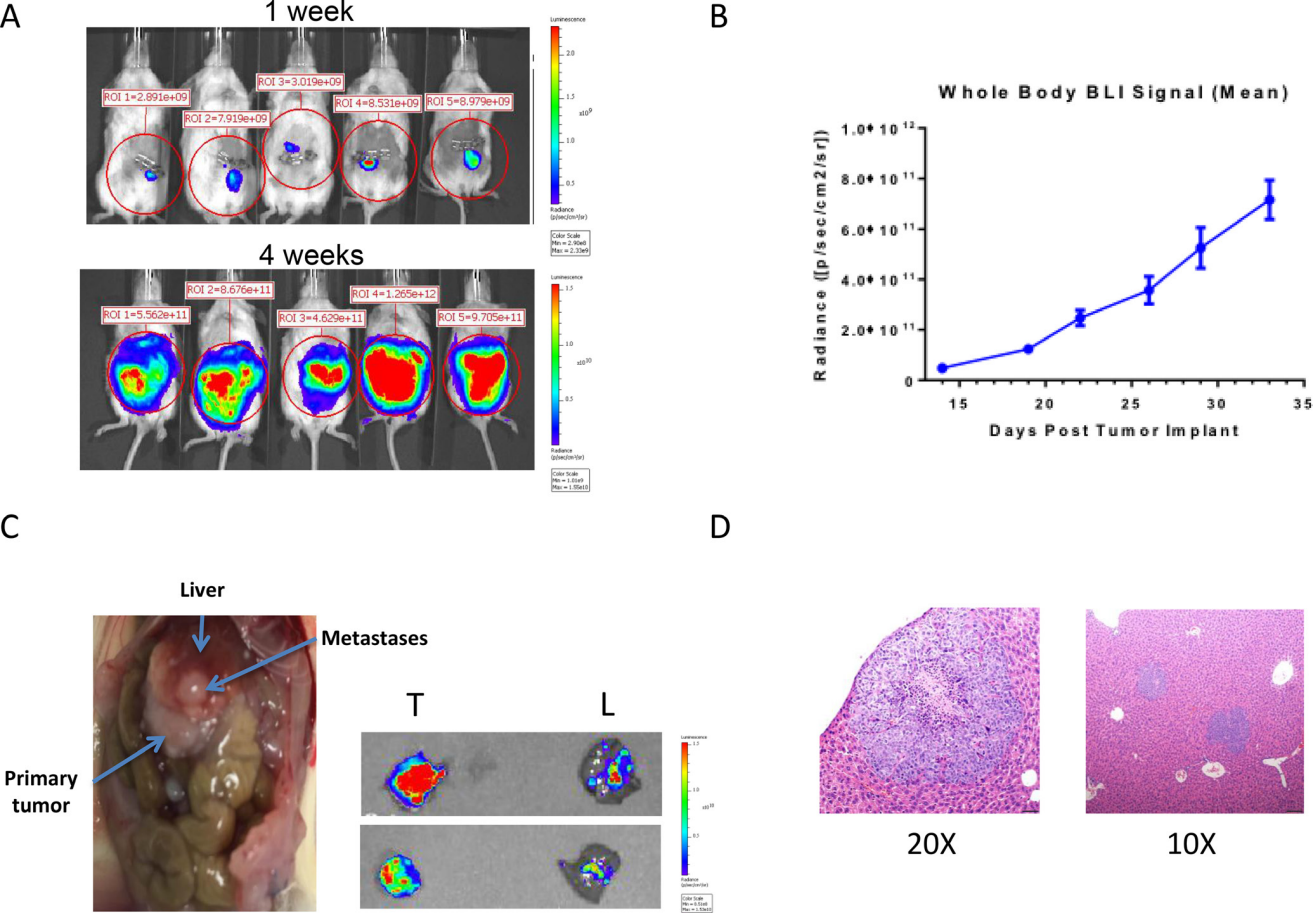
Development of a CRC PDX model of spontaneous metastasis to the liver **(A)** Luc2-mCherry tagged UM-CRM 12-1159 tumor spheres were orthotopically implanted into the cecum, followed by bioluminescent monitoring of tumor burden by whole body imaging. **(B)** Time-dependent increase in whole body bioluminescence following tumor implantation of Luc2-mCherry tagged cells (*n* = 11–14 for all time points except D29 and D33, where *n* = 4). **(C)** Macroscopic evidence of abdominal metastases in necropsied animals at the time of death. *Ex vivo* imaging of primary tumors (T) and livers (L) was carried out on two representative animals at the time of death. **(D)** H & E staining was carried out on liver sections to obtain evidence of intrahepatic metastasis, with CRC metastatic cell populations in the liver showing more intense hematoxylin (purple) staining.

To further define the therapeutic efficacy of MEDI3622 in a potentially more biologically relevant pre-clinical model of advanced CRC disease in patients, we developed an orthotopic, PDX metastatic model of colon cancer. CRM 12-1159 PDX CRC cells modified to express a luciferase and mCherry fluorescence reporter construct (CRM 12-1159 Luc2-mCherry cells) were orthotopically implanted into the cecum, and bioluminescence was assessed over time to monitor relative tumor burden at the primary site and distant organs. A time-dependent increase in bioluminescent signal was observed non-invasively (Figure 4A, 4B). *Ex vivo* evaluation of bioluminescence was carried out on organs excised from mice that were euthanized, when the mice began to exhibit signs of the effects of their tumor burden (hunched posture, body weight loss, rough pelage), generally occurring at five weeks following implantation of the primary tumor (Figure 4C). At that time, all of the mice had enlarged tumors in the cecum, as well as macroscopic metastatic lesions on the liver. Histological examination of the liver of the mice showed that metastatic growth was occurring under the capsule that surrounds the liver, confirming that the tumors arose through tumor cells seeded through the vascular system, rather than adhesion of cells to the outside of the liver (Figure 4D).

**Figure 5:**
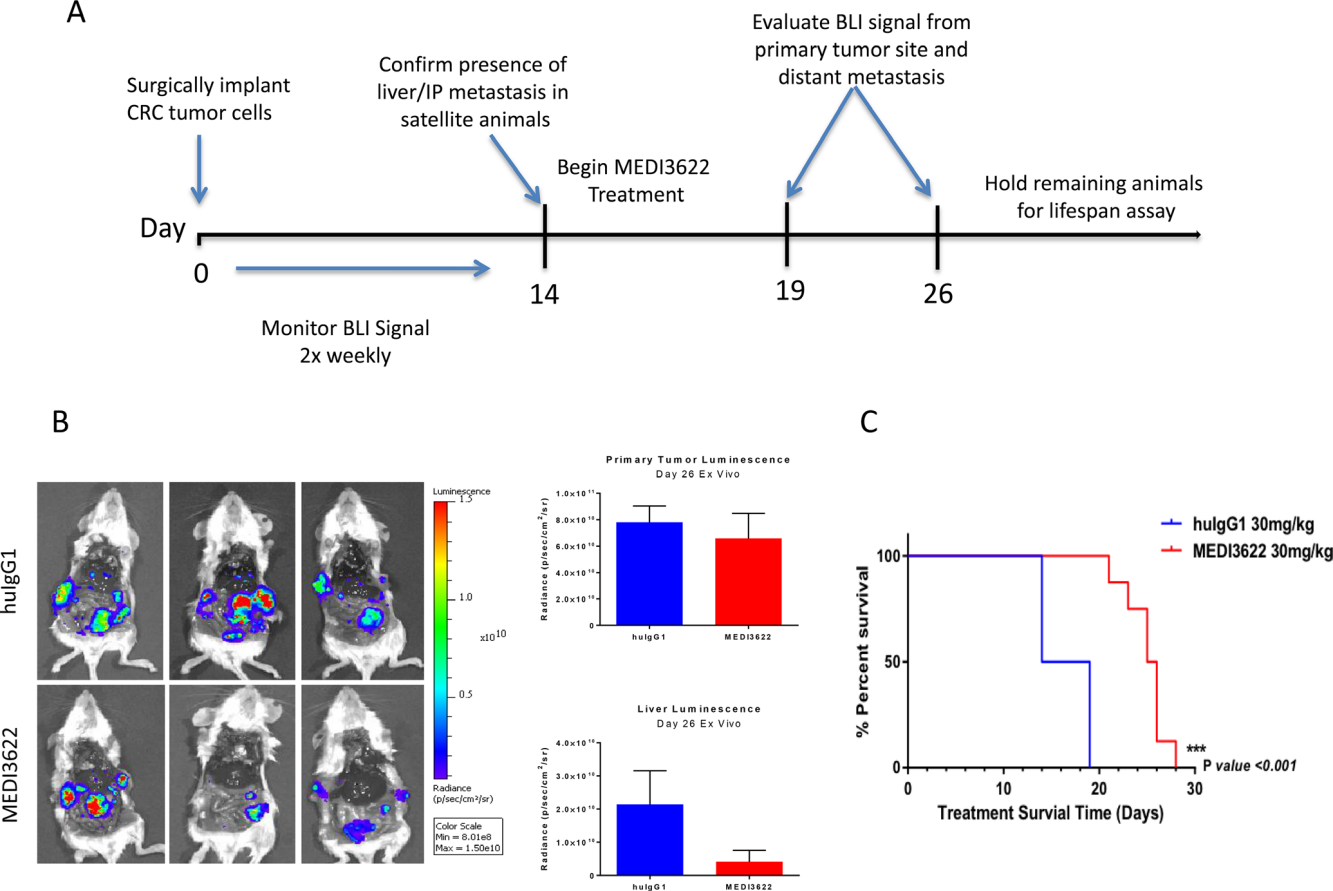
Evaluation of the efficacy of MEDI3622 and its impact on survival of animals orthotopically implanted with UM-CRM 12-1159 tumors **(A)** Study schema for evaluation of MEDI3622 to assess its effects on metastatic CRC in the PDX model. **(B)** *Ex vivo* imaging after two treatment cycles (2 weeks). Bioluminescence was quantitated in the excised primary tumors and livers as shown in the bar chart on the right (*n* = 3). **(C)** Meier-Kaplan survival analysis of mice orthotopically implanted with UM CRM-12-1159 tumors treated with either vehicle (blue) or MEDI3622 (red) for two treatment cycles (*n* = 14).

The efficacy of MEDI3622 against orthotopically implanted CRM 12-1159 tumor cells was subsequently evaluated. Study design is depicted in Figure 5A. Whole body bioluminescence imaging (BLI) was carried out non-invasively twice weekly for two weeks following primary tumor implantation. On day 14, three mice were euthanized and the presence of liver metastasis was confirmed by bioluminescence assessment of excised tissues. Mice were then randomized into two groups receiving either huIgG1 control or MEDI3622 twice weekly for the remainder of the experiment. Included within each treatment group were extra satellite mice, to be euthanized for *ex vivo* BLI after the first and second cycle of treatment. *Ex vivo* evaluation of MEDI3622-treated mice showed a significant reduction in bioluminescence in the liver but not in the primary tumor (Figure 5B). Since metastatic lesions in the liver were shown to be present before treatment was initiated, this result suggests that MEDI3622 is efficacious against disseminated disease. The modest reduction in bioluminescence at the primary site of orthotopic implantation may reflect limitations in drug penetration. Importantly, treatment with MEDI3622 led to a statistically significant increase in life span of 9 days (Figure 5C).

## DISCUSSION

We report here that MEDI3622, a highly specific and potent anti-ADAM17 monoclonal antibody, impairs growth of multiple CRC PDX models. ADAM-17, which has been implicated in the proteolytic cleavage of EGFR ligands, among other activities, has been shown to be upregulated in CRC tumor specimens compared with ADAM17 expression levels in normal colonic mucosa [14]. The previously reported efficacy of MEDI3622 against Colo 205 xenografts provided the impetus for its broader evaluation in a genomically heterogeneous panel of CRC PDX models. Colo 205 tumors, which are known to express ADAM17, do not exhibit shedding of ADAM17-dependent HER pathway ligands and are resistant to HER-based therapies [9]. Therefore, the potential of this agent to elicit HER-independent activity was of particular interest to explore in the CRC setting, where patients often exhibit resistance to cetuximab and other EGFR or HER2 targeted agents.

In the present study, three of the five CRC PDX models tested proved to be responsive to MEDI3622 treatment. Consistent with the expectation that activity of this agent against CRCs would prove to be HER-independent, we found no correlation between basal expression of activated EGFR in the various models and their sensitivity to MEDI3622. However, we cannot rule out that inhibition of HER ligand shedding by MEDI3622 contributes to its overall activity in some models, such as CRM 12-1159, one of the two most sensitive models to MEDI3622, which exhibited significantly higher levels of phosphorylated EGFR compared to all of the other models tested in this study (Supplementary Figure 3).

Others who have investigated the consequences of targeting ADAM17 in pre-clinical CRC models have reported that ADAM17 regulates chemotherapy-induced activation of EGFR and ligand shedding, irrespective of the mutational status of KRAS [15]. Our data with MEDI3622 showing activity in a KRAS^MT^ tumor supports further testing in an expanded cohort of KRAS^MT^ CRC models to explore its potential therapeutic utility across a wide spectrum of colorectal cancers. Efforts are underway to identify molecular markers shared by MEDI3622-sensitive CRC tumor models to inform clinical trial design.

MEDI3622 is generally ineffective at inhibiting the growth of CRC cells *in vitro* as reflected by IC_50_ values > 10 μM (Supplementary Figure 5). The striking contrast between *in vitro* and *in vivo* sensitivity suggests that MEDI-3622 is likely to be exerting effects on the tumor microenvironment. In addition to its effects on activation of growth factors of the EGF family, ADAM17 has been reported to induce VEGF-A synthesis to promote tumor angiogenesis [16–18]. Others have reported that ADAM17 supports processes that are central to lymphangiogenesis, such as motility, migration, and invasion of lymphatic endothelial cells [19]. However, no effects on the tumor vasculature as measured by CD31 immunostaining were observed in the MEDI3622-treated CRM 12-1159 tumors that had exhibited impaired progression (data not shown). Additionally, our antibody is a human IgG1 molecule and could therefore, work through antibody-dependent cell-mediated cytotoxicity (ADCC). While we cannot rule out this activity in our CRC models, we have observed similar anti-tumor effects of MEDI3622 when using either an IgG1 or an effector-null version (data not shown) in different model systems.

Experimental support for MEDI3622 exerting effects on the tumor microenvironment also was seen in studies carried out to explore its effects on CRC cancer stem cells (CSCs). The molecular mechanisms regulating presumptive CSC phenotypes in CRC have not been fully elucidated. However, studies from Ellis and his colleagues have established a role for ADAM17 in promoting the CSC phenotype and chemoresistance of CRC cells [13]. The induction of CSC properties by endothelial cells is thought to be dependent on the activation of Notch signaling in CRC cells through the paracrine action of the Notch ligand Jagged-1, which is produced by the endothelial cells. Jagged-1 cleavage by ADAM17 was required to promote “stemness” in CRC. Therefore, pharmacological inhibition of ADAM17 activity is a rational approach to target the activation of Notch signaling in CRC cells to impair their tumorigenicity and metastasis. Two parallel lines of evidence in the present report suggest that MEDI3622 targets the activation of Notch signaling in colorectal tumors to exert its anticancer activity. First, only the responder models exhibited decreased gene expression of Notch family members as a consequence of treatment. Second, limiting dilution assays carried out on MEDI3622 treated tumors revealed a significant decrease in their tumor-initiating cell frequency which are dependent on Notch signaling.

The ultimate therapeutic promise of any new experimental agent being developed for the treatment of metastatic CRC will depend on its ability to impair progression of previously disseminated disease at the time of diagnosis. We report here the development of an imaging-enabled model of CRC spontaneous metastasis that allowed us to evaluate efficacy of MEDI3622 on metastatic CRC lesions arising in the liver after orthotopic implantation in the cecum. Treatment was not initiated until metastasis had been confirmed. It is encouraging that treatment with MEDI3622 conferred a 9-day increase in survival in mice implanted with UM-CRM 12-1159 tumors, an aggressive PDX model that resulted in death of all of the control mice within 20 days of surgery. Since secondary surgeries to remove the orthotopic tumors are problematic, the increase in survival arising as a consequence of MEDI3622 treatment could be a function of the activity of this agent not only on metastatic lesions present at the time treatment was initiated, but also on the formation of new metastases. A prometastatic role for Notch signaling in CRC was recently reported, revealing a previously unknown connection between NOTCH and RHO signaling to regulate CRC invasion and metastasis [20, 21]. Genomic and phosphoproteomic studies that are underway should shed light on whether MEDI3622 sensitive CRC models show any evidence of aberrant RHO signaling.

In summary, we have demonstrated that targeting of ADAM17 with the antibody MEDI3622 significantly impairs tumor growth in multiple CRC PDX models. CRC CSC function was inhibited by MEDI3622 treatment, as reflected by reduction in tumor initiating cell frequency in tumor re-implantation studies at the conclusion of treatment. MEDI3622 was further found to inhibit growth of metastatic lesions arising in the liver after orthotopic implantation leading to increased survival. The promising preclinical activity seen here supports further evaluation of this treatment approach to improve therapeutic outcome for patients diagnosed with metastatic colorectal cancer.

## MATERIALS AND METHODS

### Antibody generation and characterization

MEDI3622 evolved from screening of a Dyax phage display library for Fabs capable of inhibiting recombinant ADAM17 as previously described [9]. One of the hits was subsequently converted to the IgG1 antibody 80PH3, exhibiting an IC_50_ in biochemical assays of 0.46 nmol/L against ADAM17. Cell-based shedding assays were then carried out to optimize cellular potency leading to the generation of MEDI3622.

### Patient samples and establishment of patient-derived colorectal xenograft models

For establishing models designated either CRC or CRM, tumor and matched normal specimens were obtained from patients undergoing liver metastectomies or colon resections of primary disease at the University of Michigan University Hospital (Ann Arbor, MI). All patients provided informed written consent and samples were procured with approval of the University of Michigan Institutional Review Board (HUM00065489). Specimens were obtained within four hours of surgery and immediately transferred to DMEM/F12 media supplemented with 10% fetal bovine serum and 1% Penicillin-Streptomycin-Glutamine at 4°C. A portion of normal colon specimens were fixed in 10% neutral buffered formalin (NBF) and the remainder snap frozen in liquid nitrogen. Portions of tumor specimens were either fixed in 10% NBF, snap frozen in liquid nitrogen, or divided into fragments approximately 3 × 3 mm for subcutaneous implantation into female 6–7 week old CIEA NOG mice (NOD.Cg-Prkdcscid Il2rgtm1Sug/JicTac from Taconic) using an 11G Trocar needle. Tumor-implanted mice were monitored for tumor growth for up to four months following implantation. Xenografted tumors from the NOG mice were passaged into female 6–7 week old NCR nude mice (CrTac:NCr-Foxn1nu from Taconic) for model expansion. For the models designated GB-CO, colorectal tumor samples were purchased from Asterand Biosciences and in accordance with MedImmune policies and procedures. Tumor fragments were implanted into 6–8 week old NSG mice (NOD.Cg-*Prkdcscid Il2rgtm1Wjl*/SzJ) using an 11G Trocar needle. Tumor growth was monitored. Fragments were subsequently passaged into 6–8 week old NSG mice. PDX models were maintained in nude mice for a maximum of four passages before fresh material from the freezer was used to regenerate the line.

### Xenograft efficacy studies

Female 6–7 week old NCR nude mice (CrTac:NCr-*Foxn1nu* from Taconic) were implanted subcutaneously with low passage PDX tumor fragments (∼30 mg) into the region of the right axilla. Mice were randomized into treatment groups and treatments initiated when tumors reached 100 to 200 mg. Human IgG1 control or MEDI3622 antibody was administered twice weekly via intraperitoneal injection as a solution in phosphate-buffered saline (0.2 ml/20g body weight). Tumor volume and body weights were measured two or three times a week. Tumor volumes were calculated by measuring two perpendicular diameters with calipers and using the formula: tumor volume = (length × width^2^)/2. When mean tumor burden in the IgG control group reached ∼1000 mg, mice were euthanized 72 hours following the last treatment. Following euthanasia, whole blood was collected via cardiac puncture into serum separator tubes. Whole blood was kept at room temperature for 30 minutes, centrifuged, and the serum collected and stored at -80°C until use. Tumors were harvested from mice at the time of euthanasia and divided into three pieces. The first piece was fixed in 10% neutral buffered formalin for subsequent immunohistochemical analysis. The second piece was snap frozen in liquid nitrogen and stored at –80°C until use. The third piece was minced, slowly frozen in 90% FBS/10% DMSO, and stored at −80°C until use. Percent treated/control (%T/C) was calculated by dividing the median treated tumor weight by the median control tumor weight and multiplying by 100 on the last day of treatment. All procedures related to the handling, care, and treatment of animals was conducted in accordance with University of Michigan’s Committee on the Use and Care of Animals guidelines.

### Development of an orthotopic model of CRC metastasis

A lentiviral expression vector for luminescence and fluorescence labeling of cells, Luc2-ires-mCherry, was constructed by inserting the Luc2 open reading frame for firefly luciferase, obtained from pGL4.10 [luc2] (Promega), into the MCS of the bicistronic pLVX-EF1α-IRES-mCherry lentiviral expression vector (Clontech). The resulting construct expresses firefly luciferase and mCherry as separate proteins due to the internal ribosome entry site (IRES), with very tight (> 99%) correlation between mCherry and luciferase expression as assessed by colony formation assays (data not shown). Lentiviruses were prepared from 293T cells by calcium chloride transfection with plasmids Delta and VSV (gift of D. Baltimore) and used to tranduce dissagregated primary xenografts, which were re-implanted immediately. FACS sorting of resulting bulk tumors was carried out to purify the mCherry-positive population prior to subcutaneous re-implantation. Resulting tumors were dissociated and the luciferase- and mCherry-tagged tumorsphere line was propagated *in vitro* and expanded to generate sufficient tumorsphere material for orthotopic studies.

On the day of orthotopic implantation, tumorspheres were dissociated and diluted in a 1:1 mixture of DMEM/F12 and Matrigel. Cell mixtures were absorbed into 3 × 3 × 1 mm pieces of Gelfoam^®^ absorbable gelatin sponge (Pfizer Injectables) so that each Gelfoam^®^ piece contained a final concentration of 5 × 10^5^ cells. Laparotomies were performed to exteriorize the cecum and ascending colon. Using magnification and microsurgery techniques, the serosa of CIEA NOG mice were disrupted and a piece of the Gelfoam^®^ was positioned and sutured to the submucosal layer of the cecum. The bowel was then returned to the peritoneal cavity and the abdomen closed with sutures. Bioluminescent imaging was performed using an IVIS 200 device (Caliper Life Sciences) to monitor growth of the primary mass and metastatic spread to the liver and other organs.

### Limiting dilution assays

Viable and lineage depleted cells were re-suspended in DMEM/F12 and Matrigel (BD Biosciences). Cells were injected subcutaneously in female 6–7 week old NCR mice at the indicated number of cells per injection. Animals were monitored by palpation twice weekly for at least 10 weeks following injection to detect tumor formation. Tumors were counted as detectable at 50 mm^3^ in calculated size using the formula: tumor volume = (length × width^2^)/2.

### TACE activity assays

Pharmacodynamic activity of MEDI3622 in excised tumors was measured by testing lysates in a fluorometric assay to measure hydrolysis of a FRET-tagged substrate by TACE (Sigma-Aldrich MAK218).

### Quantitative reverse-transcription (RT) PCR

Total RNA was extracted from tumors using RNeasy Plus Mini Kit (Qiagen). Each RNA sample was reverse-transcribed using High Capacity cDNA Reverse Transcription Kit (Applied Biosystems). The resulting cDNAs were used as starting material for singleplex real-time PCR using Taqman gene expression assays JAG1 (Hs01070032_m1), JAG2 (Hs00171432_m1), HES1 (Hs00172878_m1), HES2 (Hs01021800_g1), HEY1 (Hs01114113_m1), HEY2 (Hs00232622_m1) and GAPDH (Hs02758991_g1) according to the manufacturer’s protocol (Applied Biosystems). Quantitative gene expression analyses were performed using the ViiA7 Real-time PCR System (Applied Biosystems). Data were calculated as DC_t_ = Ct ^notch-related gene^ – C_t_^GAPDH^. Results of tumors treated with MEDI3622 were normalized to IgG control-treated tumors.

### Statistical analysis

For limiting dilution analysis, the frequency of tumorigenic cells and the 95% confidence interval were calculated using extreme limiting dilution software (http://bioinf.wehi.edu.au/software/elda/). For *in vivo* efficacy and life span studies, analysis was carried out using log-rank test and *t*-test using Prism Software (GraphPad).

## SUPPLEMENTARY MATERIALS FIGURES AND TABLES


