# Supplementary material for: Targeting ADAM17 inhibits human colorectal adenocarcinoma progression and tumor-initiating cell frequency
Source: Oncotarget. 2017 May 10;8(39):65090–9. doi: 10.18632/oncotarget.17780 (PMC5630314; doi:10.18632/oncotarget.17780)
Supplement: Supplementary file 1 [file oncotarget-08-65090-s001.pdf]

Targeting ADAM17 inhibits human colorectal adenocarcinoma progression and tumor-initiating cell frequency

SUPPLEMENTARY MATERIALS

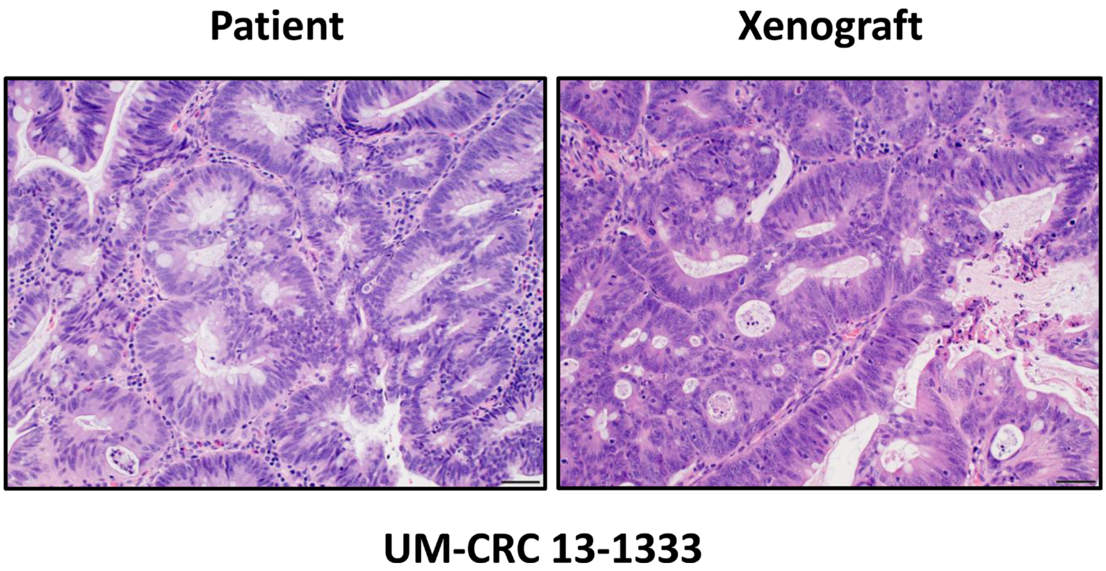

Supplementary Figure 1: Histology of a representative primary tumor xenograft model (CRC 13-1333) and the patient tumor from which it was derived.

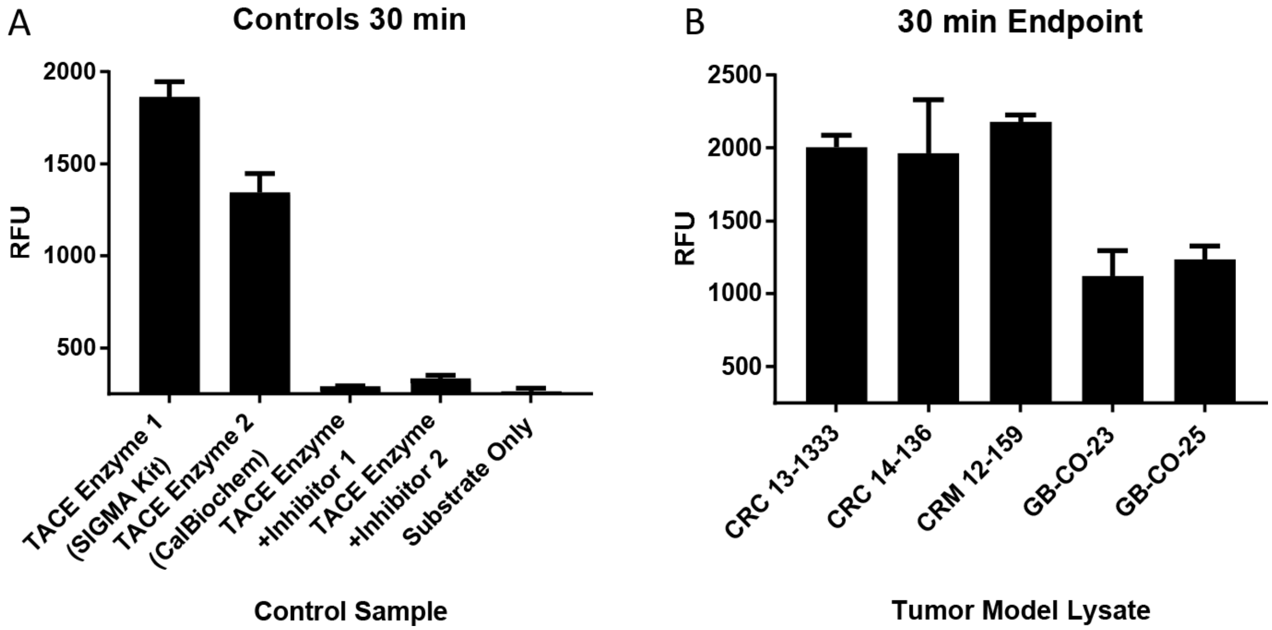

Supplementary Figure 2: TACE activity measurements. Panel (**A**) shows enzyme activity of a recombinant, exogenously added TACE enzyme from two different sources along with the inhibitors supplied by the kit as well as background readings of substrate only. Panel (**B**) is the activity measured in the 5 CRC PDX models used in this report.

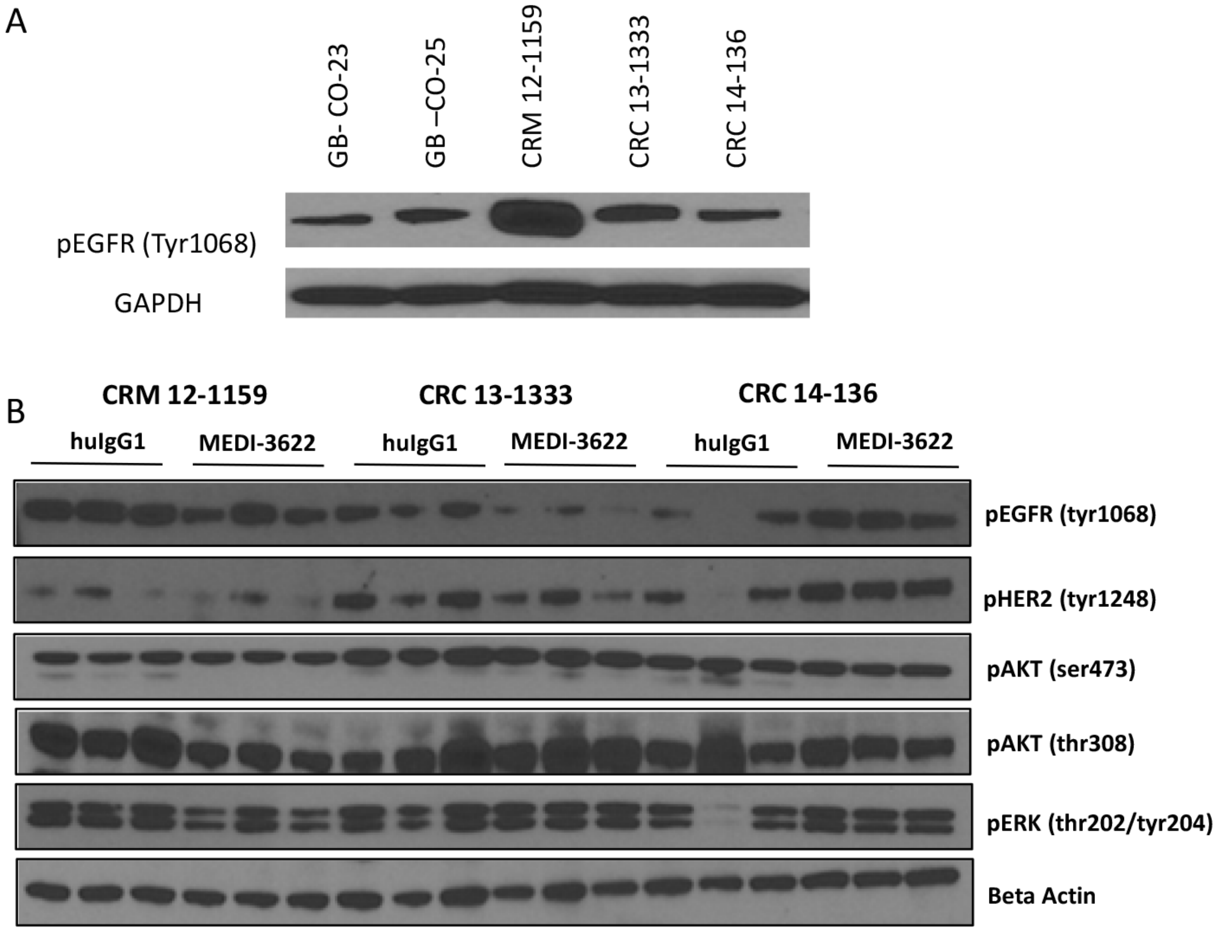

Supplementary Figure 3: (**A**) Immunoblotting analysis of pEGFR (Tyr1068) expression in the five CRC patient-derived xenograft models studied in this report. (**B**) Immunoblotting of pEGFR, pHER2, pAkt and pERK from tumors excised from animals treated with either the control IgG1 or MEDI3622.

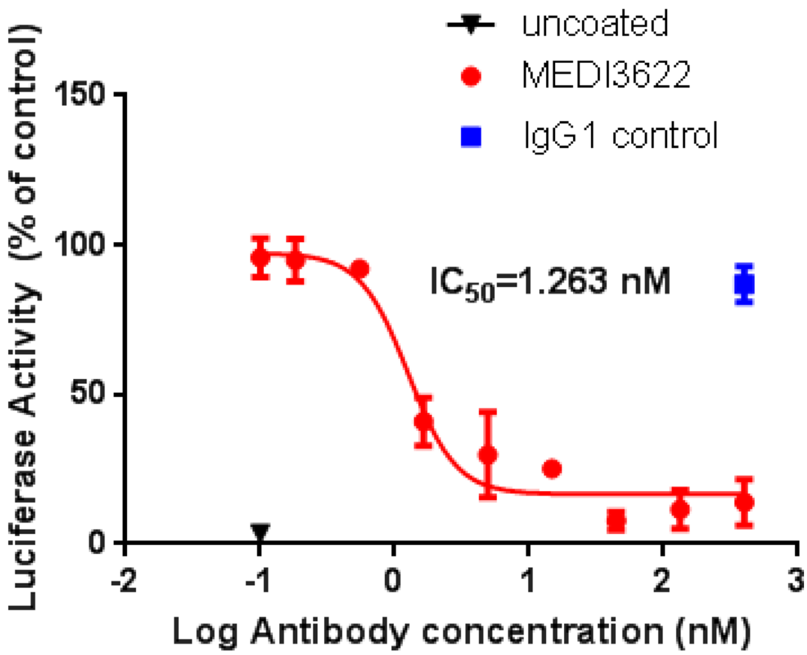

Supplementary Figure 4: Notch (RBP-Jk) luciferase activity assay in the presence of increasing amounts of MEDI3622. Luciferase activity in the absence of ligand (DLL4) is shown by the black triangle and the IgG1 control at the highest concentration is shown by the blue square.

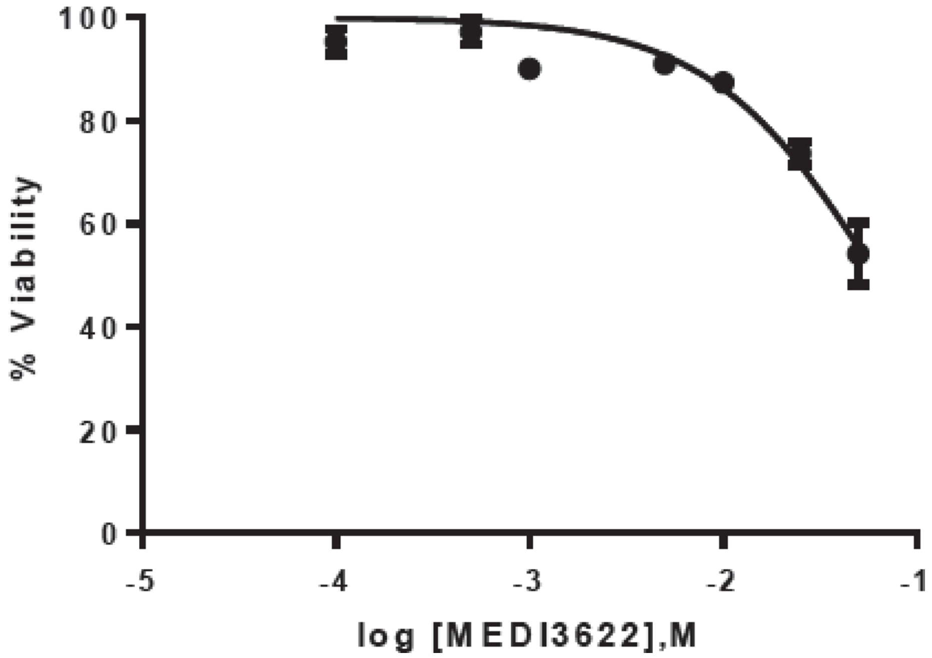

Supplementary Figure 5: *In vitro* sensitivity of UM-CRM 12-1159 tumorspheres treated for 48 h with varying concentrations of MEDI3622.

Supplementary Table 1: Selected clinical, pathological, and genomic features in the CRC patients and the tumors from which the respective PDX models were derived

Model	Gender	Age	Prior Chemotherapy	Location	Histopathology				Mutational Status		
					Type	TNM	Stage	Degree of Tumor Differentation (Grade)	KRAS	BRAF	PIK3CA
GB-CO-23	F	86	No	Right colon	ADC	pT3N0	IIA	Moderate	G12V	WT	E545K
GB-CO-25	F	79	No	Right colon	ADC	pT3N0	IIA	Moderate	WT	WT	WT
UM-CRM 12-1159	M	51	Unkn	Liver	ADC	Unkn	Unkn	Moderate	WT	WT	H1047R
UM-CRC 13-1333	F	53	No	Sigmoid colon	ADC	pT3N1b	IIIB	Moderate	G12D	WT	Q546L
UM-CRC 14-136	M	54	No	Right colon	MUC	pT3N0	IIA	Moderate	Q61H	WT	E542K

Supplementary Table 2: Details of the TIC analysis shown in Figure 3. The numbers of cells and observed take rates are reported along with the TIC frequency and range calculated by the L-calc software

UM-CRC-13-1333

Treatment Group	Number of cell implanted			TIC Frequency (95% CI)	TIC Range
	100	1000	10000		
	Tumor formation/number of animals implanted				
huIgG1	0/5	1/5	3/5	1:9342	1:5494 to 1:15,889
MEDI3622	0/5	0/5	2/5	1:22,379	1:11,059 to 1:45,284

Colo 205

Treatment Group	Number of cells implanted					TIC Frequency (95% CI)	TIC Range
	50	100	250	500	1000		
	Tumor formation/number of animals implanted						
huIgG1	7/10	9/10	10/10	9/9	10/10	1:42	1:32 to 1:55
MEDI3622	5/10	6/10	9/10	10/10	10/10	1:96	1:62 to 1:102

GB-CO-23

Treatment Group	Number of cells implanted					TIC Frequency (95% CI)	TIC Range
	100	500	1000	5000	10000		
	Tumor formation/number of animals implanted						
huIgG1	0/5	0/5	0/5	2/5	3/5	1:15,953	1:9,722 to 1:26,177
MEDI3622	0/5	0/5	0/5	0/5	4/5	1:15,206	1:9,349 to 1:24,733
